# Transmitted drug resistance in recently infected HIV-positive Individuals from four urban locations across Asia (2007–2010) – TASER-S

**DOI:** 10.1186/s12981-015-0043-1

**Published:** 2015-02-13

**Authors:** Awachana Jiamsakul, Sunee Sirivichayakul, Rossana Ditangco, Ka-Hing Wong, Patrick CK Li, Jutarat Praparattanapan, Praphan Phanuphak, Edelwisa Segubre-Mercado, Wing-Cheong Yam, Thira Sirisanthana, Thida Singtoroj, Matthew Law, on behalf of the TREAT Asia Studies to Evaluate Resistance – Surveillance Study (TASER-S)

**Affiliations:** The Kirby Institute, UNSW Australia, Sydney, Australia; Faculty of Medicine, Chulalongkorn University and HIV-NAT/The Thai Red Cross AIDS Research Centre, Bangkok, Thailand; Research Institute for Tropical Medicine, Manila, Philippines; Integrated Treatment Centre, Hong Kong, China; Department of Medicine, Queen Elizabeth Hospital, Hong Kong, China; Research Institute for Health Sciences, Chiang Mai University, Chiang Mai, Thailand; Department of Microbiology, Queen Mary Hospital, Faculty of Medicine, The University of Hong Kong, Hong Kong, China; TREAT Asia, amfAR - The Foundation for AIDS Research, Bangkok, Thailand

**Keywords:** Transmitted, Drug resistance, Asia, Resource-limited, Recent-infection

## Abstract

**Background:**

The availability of HIV antiretroviral therapy (ART) has been associated with the development of transmitted drug resistance-associated mutations (TDRM). TDRM can compromise treatment effectiveness in patients initiating ART and the prevalence can vary in different clinical settings. In this study, we investigated the proportion of TDRM in treatment-naïve, recently infected HIV-positive individuals sampled from four urban locations across Asia between 2007–2010.

**Methods:**

Patients enrolled in the TREAT Asia Studies to Evaluate Resistance – Surveillance Study (TASER-S) were genotyped prior to ART initiation, with resulting resistance mutations analysed according to the WHO 2009 list.

**Results:**

Proportions of TDRM from recently infected individuals from TASER-S ranged from 0% to 8.7% - Hong Kong: 3/88 (3.4%, 95% CI (0.71%-9.64%)); Thailand: Bangkok: 13/277 (4.7%, 95% CI (2.5%-7.9%)), Chiang Mai: 0/17 (0%, 97.5% CI (0%-19.5%)); and the Philippines: 6/69 (8.7%, 95% CI (3.3%-18.0%)). There was no significant increase in TDRM over time across all four clinical settings.

**Conclusions:**

The observed proportion of TDRM in TASER-S patients from Hong Kong, Thailand and the Philippines was low to moderate during the study period. Regular monitoring of TDRM should be encouraged, especially with the scale-up of ART at higher CD4 levels.

## Background

Since 2001, the World Health Organization (WHO) has been promoting a public-health approach to improve access to antiretroviral therapy (ART) in resource-limited settings [1]. With the scale up of ART, patients who experienced virological failure or those who failed to suppress the virus may continue to have ongoing viral replication under drug pressure which increases the risk of the development of resistance-associated mutations (RAMs). These RAMs may further compromise future treatment outcomes and can be transmitted, leading to primary drug resistance in other un-treated individuals [2,3].

In resource-limited countries in Asia, non-nucleoside reverse transcriptase inhibitors (NNRTI) is predominantly used in standard first-line therapy. A Thai study reported a 2% prevalence of transmitted drug resistance-associated mutations (TDRM) in recently HIV-infected patients, with the annual proportion rising from 0% to 5.2% between 2003–2006. The most common RAMs found in this study were M184V and Y181C reflecting lamivudine and NNRTI resistance [4]. Hong Kong SAR China, a high-income locality, reported a TDRM prevalence of 3.6% between 2003–2007, with the protease inhibitors (PI) RAMs (M46I/L and L33F) being the most common mutations consistent with widespread use of PI-based initial regimen [5]. Conversely, an Australian study found rates of TDRM to have dropped dramatically after the introduction of combination ART in 1996 [6].

The objective of this study was to evaluate the extent of TDRM in treatment-naïve, recently infected HIV-positive individuals in selected Therapeutics, Research, Education, and AIDS Training in Asia (TREAT Asia) sites, and to determine changes in proportion of TDRM over time. The reported findings may not be representative of the broader Asia-Pacific region, but rather of those who participated in the study.

## Methods

Treatment-naïve, recently HIV-infected patients were recruited into the TREAT Asia Studies to Evaluate Resistance – Surveillance Study (TASER-S) between 2007-2010 [7] from 5 participating sites: 2 from Hong Kong SAR, China; 1 from Bangkok, Thailand; 1 from Chiang Mai, Thailand; and 1 from Manila, Philippines. Patients were included in TASER-S according to the following inclusion criteria:

***Hong Kong SAR, China:*** patients were selected from those who attended the two participating clinics and satisfied one of these criteria: (a) age up to 25 years old; (b) negative HIV test within one year; or (c) BED assay positive.

***Bangkok, Thailand:*** patients were enrolled from a voluntary counselling and testing centre in Bangkok. All modes of HIV exposure were considered, however male patients with homosexual HIV exposure were selectively chosen to be enrolled in 2010. Recent HIV infection was defined as a new HIV diagnosis in subjects <25 years old. For those who were older, previous HIV-negative documentation within the past 12 months was required for study inclusion.

***Chiang Mai, Thailand:*** patients presented to care at the participating hospital were selected for enrolment. Patients were included if they had a confirmed HIV-positive test and were aged <25 years with no prior AIDS-defining illnesses.

***Manila, Philippines:*** blood samples were obtained from the STD AIDS Cooperative Central Laboratory, the National Reference Laboratory for HIV and Other STIs (NRL-SACCL). All samples obtained from asymptomatic patients were tested using the BED assay. Those with positive BED tests were presumed to be recently infected and included in TASER-S.

CD4 count was not used as a criteria in TASER-S as the study protocol was developed prior to the WHO 2008 surveillance recommendations [8], and it was not known a priori the time lag for obtaining CD4 results.

Genotyping was performed at TREAT Asia Quality Assessment Scheme (TAQAS) certified laboratories [9]. Pre-treatment *pol* gene FASTA files were submitted to the Stanford University HIV Drug Resistance Database [10] Version 6.2 for genotyping and REGA HIV-1 Subtyping Tool [11,12] - Version 2.0 for subtyping. RAMs were analysed according to the WHO 2009 list [13]. TDRM was defined as having ≥1 RAM. Patients with both protease (PR) and reverse transcriptase (RT) sequences available were included.

Clinical characteristics, including age, sex, mode of HIV exposure, viral load, CD4 count and HIV-1 subtype, were reported descriptively. Time trends were analysed using chi-squared test for trend. Confidence intervals (CI) for proportion of RAMs were calculated using the exact binomial methods. A sensitivity analysis was performed by including patients who were missing a PR or RT sequence file by assuming an absence of RAMs in the missing gene region and also by including those with extensive RAMs without confirmation of their treatment naïve status. All data management and statistical analyses were performed using SAS software version 9.3 (SAS Institute Inc., Cary, NC, USA) and STATA software version 12.1 (STATA Corp., College Station, TX, USA).

Data transfers were aggregated at The Kirby Institute, UNSW Australia. Ethics approvals were obtained from UNSW Australia Ethics Committee and institutional review boards at the participating clinical sites and coordinating centre (TREAT Asia/amfAR, Bangkok, Thailand). Written informed consent was obtained from participants prior to enrolment – except in the Philippines, where anonymous samples were obtained from the NRL-SACCL and consent was waived.

## Results

### Hong Kong SAR, China

**Figure 1:**
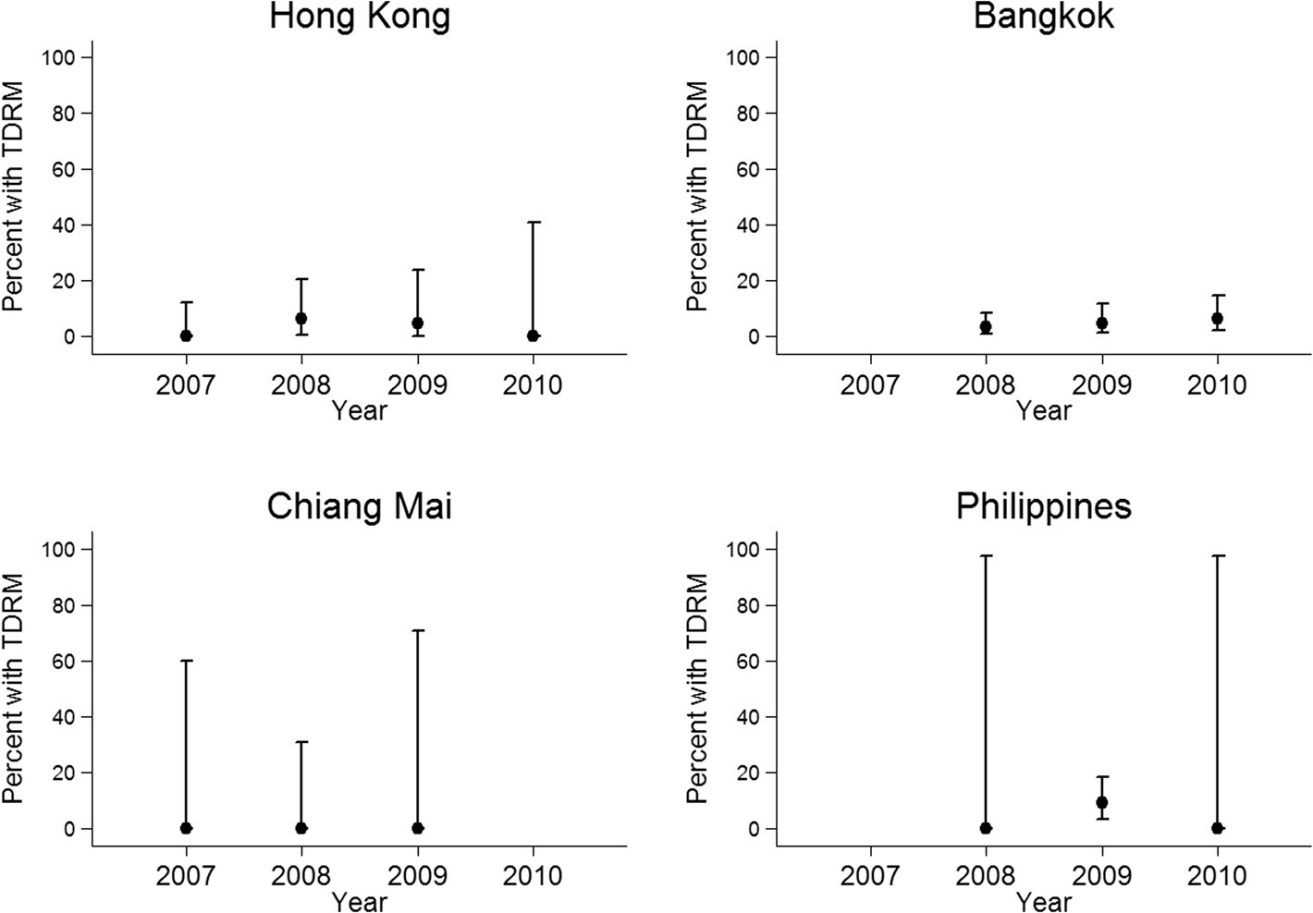
**Proportion of transmitted drug-resistance mutations (TDRM).**

A total of 88 patients were included from the 2 sites (Table 1). The overall proportion was 3/88 (3.4%, 95% CI (0.71%-9.64%)). Figure 1 shows TDRM in 2007 was 0/28 (0.0%); 2008: 2/32 (6.3%); 2009: 1/21 (4.8%); and 2010: 0/7 (0.0%), p-trend = 0.631.

**Table 1:** **Patient characteristics**

	**Hong Kong**		**Bangkok, Thailand**		**Chiang Mai, Thailand**	**Manila, Philippines**	
	**Total = 88 (%)**		**Total = 277 (%)**		**Total = 17 (%)**	**Total = 69 (%)**	
	**Without TDRM = 85 (96.6)**	**With TDRM = 3 (3.4)**	**Without TDRM = 264 (95.3)**	**With TDRM = 13 (4.7)**	**Without TDRM = 17 (100.0)**	**Without TDRM = 63 (91.3)**	**With TDRM = 6 (8.7)**
**Age (years)**	**Median = 29**	**Median = 42**	**Median = 23**	**Median = 23**	**Median = 22**	**Median = 25**	**Median = 30.5**
	IQR (24–37)	IQR (32–56)	IQR (21–24)	IQR (20–25)	IQR (21–23)	IQR (23–28)	IQR (25–33)
<25	25 (29.4)	0 (0.0)	207 (78.4)	9 (69.2)	17 (100.0)	28 (44.4)	1 (16.7)
25-34	30 (35.3)	1 (33.3)	47 (17.8)	4 (30.8)	0 (0.0)	34 (54.0)	4 (66.7)
35-44	21 (24.7)	1 (33.3)	7 (2.7)	0 (0.0)	0 (0.0)	1 (1.6)	1 (16.7)
≥45	9 (10.6)	1 (33.3)	3 (1.1)	0 (0.0)	0 (0.0)	0 (0.0)	0 (0.0)
**Sex**							
Male	81 (95.3)	3 (100.0)	247 (93.6)	13 (100.0)	12 (70.6)	54 (85.7)	5 (83.3)
Female	4 (4.7)	0 (0.0)	17 (6.4)	0 (0.0)	5 (29.4)	9 (14.3)	1 (16.7)
**HIV exposure**							
Heterosexual contact	9 (10.6)	0 (0.0)	30 (11.4)	1 (7.7)	10 (58.8)	9 (14.3)	1 (16.7)
Homosexual contact	72 (84.7)	3 (100.0)	229 (86.7)	11 (84.6)	7 (41.2)	39 (61.9)	3 (50.0)
Bisexual	4 (4.7)	0 (0.0)	4 (1.5)	1 (7.7)	0 (0.0)	0 (0.0)	0 (0.0)
Injecting drug use	0 (0.0)	0 (0.0)	1 (0.4)	0 (0.0)	0 (0.0)	0 (0.0)	0 (0.0)
Other/Unknown	0 (0.0)	0 (0.0)	0 (0.0)	0 (0.0)	0 (0.0)	2 (3.2)	0 (0.0)
**Viral load (copies/ml)**	Median = 77000	Median = 46000	Median = 41790	Median = 170000	Median = 75001	N/A	N/A
	IQR (20261–220000)	IQR (59000–68000)	IQR (16208–130000)	IQR (17414–360000)	IQR (39375–80119)		
≤50000	36 (42.4)	2 (66.7)	144 (54.5)	6 (46.2)	7 (41.2)	0 (0.0)	0 (0.0)
50001-250000	28 (32.9)	1 (33.3)	84 (31.8)	1 (7.7)	10 (58.8)	0 (0.0)	0 (0.0)
250001+	21 (24.7)	0 (0.0)	35 (13.3)	6 (46.2)	0 (0.0)	0 (0.0)	0 (0.0)
Missing	0 (0.0)	0 (0.0)	1 (0.4)	0 (0.0)	0 (0.0)	63 (100.0)	6 (100.0)
**CD4 (cells/uL)**	Median = 408.5	Median = 348	Median = 342	Median = 370.5	Median = 308	N/A	N/A
	IQR (267–552)	IQR (315–641)	IQR (255–479)	IQR (271–438)	IQR (103–405.5)		
≤50	4 (4.7)	0 (0.0)	5 (1.9)	0 (0.0)	4 (23.5)	0 (0.0)	0 (0.0)
51-100	1 (1.2)	0 (0.0)	6 (2.3)	0 (0.0)	0 (0.0)	0 (0.0)	0 (0.0)
101-200	4 (4.7)	0 (0.0)	20 (7.6)	1 (7.7)	1 (5.9)	0 (0.0)	0 (0.0)
201-500	44 (51.8)	2 (66.7)	128 (48.5)	8 (61.5)	10 (58.8)	0 (0.0)	0 (0.0)
>500	25 (29.4)	1 (33.3)	46 (17.4)	1 (7.7)	1 (5.9)	0 (0.0)	0 (0.0)
Missing	7 (8.2)	0 (0.0)	59 (22.3)	3 (23.1)	1 (5.9)	63 (100.0)	6 (100.0)
**Subtype**							
CRF01_AE	24 (28.2)	0 (0.0)	218 (82.6)	11 (84.6)	14 (82.4)	22 (34.9)	2 (33.3)
B	52 (61.2)	3 (100.0)	22 (8.3)	1 (7.7)	2 (11.8)	24 (38.1)	3 (50.0)
Other	9 (10.6)	0 (0.0)	24 (9.1)	1 (7.7)	1 (5.9)	17 (27.0)	1 (16.7)

Table 2 shows a list of RAMs harboured by different individuals. Of the 3 patients with RAMs from Hong Kong, 1 patient had a PR RAM (M46I) and 2 patients had one RT RAM each (K103N and M41L).

**Table 2:** **Protease (PR) and Reverse Transcriptase (RT) Mutations**

**Site**	**Patient**	**PR mutations**	**RT mutations**
**Hong Kong SAR, China**	1	M46I	
	2		K103N
	3		M41L
**Bangkok, Thailand**	4	M46I + V82A	
	5	M46I	
	6	M46I	
	7	L24I	
	8		T215D
	9	V82F	
	10		K103N
	11		K103N
	12		Y181C + T215I + K219E
	13	M46I + I54V + I84V + L90M	M184V
	14	M46I	Y181C + T215F
	15		Y181C + M184V
	16	M46I	
	a		D67G + K70R + K103N + Y181C + M184V + T215I + K219E
	b		L74V + F116Y + Q151M + Y181C + M184I + G190A
	c		M41L + T69D + V75M + Y181C + M184V + T215Y
**Manila, Philippines**	17	L23I	
	18		K70R
	19	I84V	
	20	G73S + N88D	
	21		M41L
	22	L23I	
	d		V106A

Note: patients a, b, c and d were those included in the sensitivity analysis for Bangkok, Thailand and Manila, Philippines.

### Bangkok, Thailand

Recruitment occurred between 2008–2010 with a total of 277 patients. TDRM was present in 13/277 (4.7%, 95% CI (2.5%-7.9%)). The proportions of TDRM by year were: 2008: 4/118 (3.4%); 2009: 4/83 (4.8%); and 2010: 5/76 (6.6%), p-trend = 0.305 (Figure 1). The most common PR-RAM was M46I (6 patients) and the most common RT-RAM was Y181C (3 patients) (Table 2).

### Chiang Mai, Thailand

Seventeen patients were enrolled into the study during the years 2007 (4 patients), 2008 (10 patients) and 2009 (3 patients). No patient harboured RAMs, with 97.5% one-sided CI of (0%-19.5%).

### Manila, Philippines

Blood samples were collected from 69 patients. TDRM was present in 6/69 patients (8.7%, 95% CI (3.3%-18.0%)). Figure 1 shows in 2008, 0/1 patients (0.0%) had TDRM; 2009: 6/67 (9.0%); and 2010: 0/1 (0.0%), p-trend >0.999.

Out of the 6 patients who harboured RAMs, all but one harboured only one resistance mutation (Table 2).

### Sensitivity analysis

With the inclusion of 5 patients with missing RT region and 3 patients with extensive mutations from Bangkok, the total proportion of TDRM for this site increased to 5.6% with 95% CI (3.2%-9.0%). The yearly trend of TDRM was not significant at p = 0.256. For Manila, the inclusion of 13 patients with missing PR or RT sequences increased the proportion to 7/82 (8.5%, 95% CI (3.5%-16.8%)), p-trend = 0.860.

## Discussion

Observed proportions of TDRM in this study ranged from 0% to 8.7%, with no significant increase in trend over time. TASER-S participants were predominantly men who have sex with men (MSM). Studies have shown that MSM was associated with the presence of TDRM with higher rates of resistance reported in this group [5,14]. Although each TASER-S site utilised a different recruitment strategy which prevents direct comparison of TDRM across sites, the findings of this study suggests that the Philippines, a country that has not yet encountered a national epidemic comparable to other Southeast Asian countries [15], had a relatively higher TDRM proportion of 8.7%. This could be due to other non-B subtypes [16] prevalent in the sample (26.1%) and may warrant further investigation into the extent of HIV RAMs within the country and the associated key population at risk.

The proportion of TDRM for Hong Kong (3.4%) and Bangkok (4.7%) were similar to that found previously [5,17] although there were differences in the sample sizes and sampling methodology. Chiang Mai reported 0% TDRM, but given that only 17 patients were enrolled, it is not possible to exclude drug resistance prevalence as high as 19.5%.

Our study had several limitations including the potential sampling bias within the study. TASER-S included only patients attending TREAT Asia sites. Informed consent was required from all participants, except in the Philippines, and this may further contribute to sampling bias by allowing only those who consented to be included in the study. Other limitations were the possibility of patients experiencing reversion of RAMs back to wild-type [18,19]; and the possibility of misclassification by BED assay due to its high false recent rates [20], although this only applies to patients recruited from Hong Kong and the Philippines. Findings of TDRM reported in this study only reflect those participating in TASER-S and may not be generalisable to the broader HIV-infected population across the region.

## Conclusions

In summary, the observed proportion of TDRM in TASER-S patients from Hong Kong, Thailand and the Philippines was low to moderate during the study period. With the 2013 WHO guidelines recommending ART initiation in persons with CD4 count ≤500 cells/μL [21], and the possible scale-up of ART due to “Test and Treat” strategies [22], further monitoring of acquired drug resistance in individuals on ART, as well as regular surveillance of recently infected persons should be encouraged.
